# A Damaged-Informed Lung Ventilator Model for Ventilator Waveforms

**DOI:** 10.3389/fphys.2021.724046

**Published:** 2021-10-01

**Authors:** Deepak K. Agrawal, Bradford J. Smith, Peter D. Sottile, David J. Albers

**Affiliations:** ^1^Department of Bioengineering, University of Colorado Denver|Anschutz Medical Campus, Aurora, CO, United States; ^2^Section of Informatics and Data Science, Department of Pediatrics, School of Medicine, University of Colorado Anschutz Medical Campus, Aurora, CO, United States; ^3^Section of Pulmonary and Sleep Medicine, Department of Pediatrics, University of Colorado Anschutz Medical Campus, Aurora, CO, United States; ^4^Division of Pulmonary Sciences and Critical Care Medicine, Department of Medicine, University of Colorado School of Medicine, Aurora, CO, United States; ^5^Department of Biomedical Informatics, Columbia University, New York, NY, United States

**Keywords:** ventilator-induced lung injury, ventilator waveform, mathematical model, acute respiratory distress syndrome, statistical inference

## Abstract

Motivated by a desire to understand pulmonary physiology, scientists have developed physiological lung models of varying complexity. However, pathophysiology and interactions between human lungs and ventilators, e.g., ventilator-induced lung injury (VILI), present challenges for modeling efforts. This is because the real-world pressure and volume signals may be too complex for simple models to capture, and while complex models tend not to be estimable with clinical data, limiting clinical utility. To address this gap, in this manuscript we developed a new damaged-informed lung ventilator (DILV) model. This approach relies on mathematizing ventilator pressure and volume waveforms, including lung physiology, mechanical ventilation, and their interaction. The model begins with nominal waveforms and adds limited, clinically relevant, hypothesis-driven features to the waveform corresponding to pulmonary pathophysiology, patient-ventilator interaction, and ventilator settings. The DILV model parameters uniquely and reliably recapitulate these features while having enough flexibility to reproduce commonly observed variability in clinical (human) and laboratory (mouse) waveform data. We evaluate the proof-in-principle capabilities of our modeling approach by estimating 399 breaths collected for differently damaged lungs for tightly controlled measurements in mice and uncontrolled human intensive care unit data in the absence and presence of ventilator dyssynchrony. The cumulative value of mean squares error for the DILV model is, on average, ≈12 times less than the single compartment lung model for all the waveforms considered. Moreover, changes in the estimated parameters correctly correlate with known measures of lung physiology, including lung compliance as a baseline evaluation. Our long-term goal is to use the DILV model for clinical monitoring and research studies by providing high fidelity estimates of lung state and sources of VILI with an end goal of improving management of VILI and acute respiratory distress syndrome.

## Introduction

Mechanical ventilation is a life-saving therapy for patients who are unable to perform gas exchange by breathing on their own. When used incorrectly, mechanical ventilation has the potential to worsen lung injury through barotrauma, volutrauma, and atelectrauma that are collectively referred to as ventilator-induced lung injury (VILI). Furthermore, while the patient and ventilator always interact, when the patient and ventilator are dyssynchronous—known as ventilator dyssynchrony (VD)—the timing and delivery of a mechanical breath in response to a patient effort may lead VILI and poor outcomes (Sottile et al., 2018a). There are conditions or syndrome such as acute respiratory distress syndrome (ARDS) that carry a high mortality rate and may be exacerbated, or even caused, by VILI (Ware and Matthay, 2000; Phua et al., 2009; Force et al., 2012; Amato et al., 2015). Therefore, identifying lung-protective ventilation to reduce VILI is both important and challenging because the oxygenation needs are often in opposition to safe ventilation, leading to a complex interplay between the underlying pulmonary pathophysiology, ventilator mechanics, and patient-ventilator interactions (Chiumello et al., 2008; Gilstrap and MacIntyre, 2013; Blanch et al., 2015; Yoshida et al., 2017). The current standard of care dictates a formulaic application of low tidal volumes to reduce overdistension and positive end-expiratory pressure to maintain patency. This approach reduces VILI but does not prevent it in all cases and is not personalized (Network, 2000; Grasso et al., 2007; Khemani et al., 2018). While such protocols have provided measurable improvements in outcomes, the formulaic approach could potentially be improved through personalization of individual respiratory mechanics and VD (Bein et al., 2013).

Modern mechanical ventilators produce data in the form of time-dependent pressure, volume, and flow waveforms that contain a wealth of information about pulmonary physiology, patient-ventilator interactions, and ventilator settings. These data can be used to troubleshoot and optimize mechanical ventilation (Corona and Aumann, 2011; Mellema, 2013). However, ventilator waveforms are typically analyzed heuristically by visual inspection and, therefore, the outcome of such an analysis is limited by individual expertise and experience (Corona and Aumann, 2011; Mellema, 2013). A quantitative interpretation of these complex signals could increase diagnostic accuracy and repeatability while facilitating the application of personalized lung-protective ventilation. One simple example of waveform quantification that is currently used in clinical care is the driving pressure, which serves as a readout of both patient condition and ventilator settings (Amato et al., 2015). In the current study, we seek to develop a model that can systematically mathematize the pathophysiologic knowledge clinicians use to interpret lung conditions from ventilator waveform data as well as knowledge about the processes governed by the ventilator.

The analysis we present herein is a departure from traditional modeling methods that link measured pressures and flows through physiologically-based parameters, such as the well-recognized single-compartment model that lumps the spatially heterogeneous lung mechanical properties into single values of resistance and compliance (Chiew et al., 2011; Hamlington et al., 2016; Mori, 2016; Mellenthin et al., 2019). In the traditional models, the entirety of the pressure and volume dynamics emerge from the hypothesized physiological mechanics. Due to this straightforward formulation, the single-compartment model is computationally efficient but often not be able to reproduce all of the features in waveform data, such as patient-ventilator interaction. This is because the model lacks the complexity to allow such complex dynamics to emerge. Given the complexity of patient-ventilator interactions and pathophysiology present in real human ventilator data, it is unlikely that a two-parameter model that does not incorporate ventilator information will be capable of representing the information that a clinician may want about lung state and pathophysiology. On the other hand, more complicated formulations, including multi-compartment models, use many states and redundant parameters that cannot be uniquely estimated, causing identifiability problems where there is no unique solution, or more often no convergent solution for parameter values. As such, those models require expensive data to estimate that are not currently available for human subjects, and require substantial computational resources. Even then, complex multi-compartment models might not produce all the relevant features present in the pressure and volume waveform data (Rees et al., 2006; Bates, 2009; Reynolds et al., 2010; Molkov et al., 2014, 2017; Roth et al., 2017; Nguyen et al., 2014; Serov et al., 2016; Ellwein Fix et al., 2018). Because of these limitations, both types of models might have a limited use in clinical settings, as the model needs to be useful for a clinician and estimable in real time.

Our approach offers the potential to overcome these limitations and provides both identifiability and fidelity by using mathematical models with interpretable parameters to recapitulate pressure and volume signals. This high fidelity is due, in part, to the limited dependence between the pressure and volume models. The relationship between components of the pressure and volume waveforms are then used to define specific physiologic features, just as the quasi-static compliance is defined from the observed ratio of tidal volume and driving pressure.

Human ventilator waveform data represent several generating processes, lung physiology, ventilator mechanics, interventions, patient-ventilator interactions, and health care process model effects (Hripcsak and Albers, 2013b; Rossetti et al., 2021). In general, physiological models alone might be missing substantial contributing sources within the data. There are many potential approaches to manage this problem. One approach would be to include models for the lungs and the ventilator to capture the mechanics of the ventilator, the lungs, and their coupled interaction. Here, instead, we are incorporating both lung and health care process model (ventilator) effects into a single unified model with targeted features captured by lumped parameters. Our model is not a mechanistic model but it is not built arbitrarily either. It is built constructively starting with a lung waveform without pathophysiology or health care processes effects (e.g., the ventilator). We added in limited, e.g., compared to a neural network or other nonlinear regression model, flexibility to the model according to features that the team deemed connected to pathophysiology or health care process model effects. The model parameters are not like Fourier components that are active during the entire breath, but rather are time-limited breath deformations that are hypothesized to relate to particular types of damage, damage inducing phenomena, and ventilator interactions and effects. It is in this way that the model is constructively anchored to physiology and health care process effects.

Therefore, in this manuscript, our objective is to build a model that takes as baseline “healthy” breaths, and then adds terms that correspond to deviations from healthy breaths whose hypothesized sources include VILI, VD, and pathophysiological features of ventilator waveforms. By estimating the model, we identify the presence and severity of deviations from normal in a way that has a physiologically-based hypothesis attached to it. In essence, this is a proof-in-principle model development manuscript with underlying constraints such that the model could potentially be of use with real clinical data. In future studies, we will tie these phenotypes to lung injury severity, VD, and the pathogenesis of VILI. We anticipate that this approach will eventually find applications in real-time clinical readouts of ventilation safety, long-term monitoring to detect changes in patient condition, and as a quantitative outcome measure for clinical trials.

In the current proof-in-principle study, our team identifies clinically important features in typical volume and pressure waveform data. We then define the models for volume and pressure waveforms as the sum of a set of terms through which we modularly capture physiologically relevant features. The pressure and volume models are coupled via the respiratory rate. This approach allows independent modeling of the waveform components so that clinical, physiologic, and ventilator-based knowledge can be used to constrain the model. We named this model the damage-informed lung-ventilator (DILV) as it contains information about both lung physiology and ventilator dynamics. To demonstrate the model’s flexibility, the volume and pressure models are qualitatively validated in a simulation study where we show various relevant features that are commonly observed in health and disease. We then identify the parameters that may correspond to interpretable pathophysiology by using the DILV model to generate pressure-volume data and qualitatively assessing the effects of parameter changes. Finally, in a quantitative verification, we demonstrate that the model can accurately and uniquely represent laboratory and clinical ventilator data, which includes mouse model and human-intensive care unit (ICU) ventilator data in the absence and presence of VD (Sottile et al., 2018a,b). Through a comprehensive comparison between the DILV model and the single-compartment model, we demonstrate that our approach can accurately determine lung compliance as a baseline evaluation. Temporal changes in the model parameters are compared to other assessments of injury severity and qualitative features of the pressure and volume waveforms.

## Materials and Methods

Mechanical ventilation is characterized by three measured state variables which vary over time: volume, pressure and flow. These time-dependent signals have diverse features arising from pulmonary physiology, the ventilator, and health care process effects such as clinical interventions, and patient-ventilator interaction (Albers and Hripcsak, 2010; Hripcsak and Albers, 2013a,b). The flow is the time-derivative of volume and so the volume variable contains the same information about the underlying lung mechanics but in a different representation (Bates, 2009). In this study, we focused on two state variables, volume and pressure.

In the simplest ventilation modes, one variable is primarily controlled by the ventilator, e.g., pressure or volume, while the other variable, e.g., volume or pressure, is free to vary, referred to as pressure-controlled ventilation (PCV) or volume-controlled (VCV), respectively. In this case, only the “free” variable contains direct information about the respiratory mechanics of the patient (Tobin, 2001; Bates, 2009). Moreover, in some models there is a rigid coupling between the controlled and free states that often limits the model flexibility, precluding the model from reproducing some features that are present in the clinical data. For example, the single-compartment model performs a linear transformation between pressure and volume variables due to the fixed coupling defined as the sum of linear resistive and elastic contributions (Bates, 2009; Smith et al., 2015; Hamlington et al., 2016). We, therefore, do not explicitly couple the controlled (also known as an independent) and free (also known as a dependent) variables such that the volume and pressure models will be independent of one another. Modern clinical ventilators also have an expansive set of other modes, the most notable of which are the patient-triggered modes where the ventilator’s action is triggered by patients such as inspiratory effort. These modes can be very lung-protective, but they can also lead to complex forms of VD that are difficult to model. Patient-triggered modes are the most commonly used modes for humans unless the human is given neuromuscular blockades (Sottile et al., 2018b).

### Identifying Important Features in the Volume and Pressure Waveform

**FIGURE 1:**
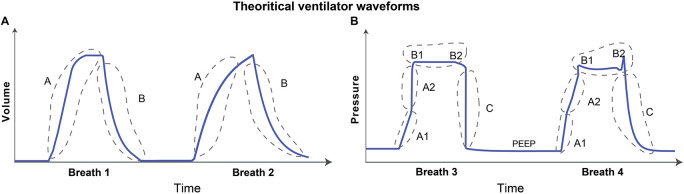
Graphical representation of theoretical volume and pressure waveforms. **(A)** The volume signal generally has two distinct features. The rising and falling of the volume signal during inspiration and expiration, respectively, are denoted as features A and B. **(B)** The pressure signal can typically have multiple features in the waveform that contain useful information. The gradient of the rising signal in which pressure continues to increase during inspiration can have two distinct features, denoted as features A1 and A2. These two features define the gradient of the rising signal before and after the inflection point such that there may be abrupt increases (breath 1) or decreases (breath 2) in the signal gradient. The shape of the plateau pressure is captured using features B1 and B2 such that there may be a peak at the beginning (B1-breath 2) and/or at the end (B2-breath 2) of the plateau. Finally, the gradient of the falling signal is captured using feature C that represents the expiration process. The baseline pressure is known as positive end-expiratory pressure (PEEP), and often used in ARDS patients to maintain an open lung (Cavalcanti et al., 2017). Note that the breaths were drawn to highlight the features which might contain useful information about lung condition and ventilator-patient interaction when the respective variable is free. If the variable is controlled, e.g., volume during VCV, the waveform features represent ventilator settings.

The volume waveform can have two characteristic features as shown schematically in Figure 1A. These features might reflect lung condition when volume is the free variable such as in PCV, otherwise these may be controlled via ventilator (Corona and Aumann, 2011; Mellema, 2013). The first feature is the inspiration, denoted as A in Figure 1A, which continues until the amount of gas delivered in that breath is reached (the tidal volume). The pressure and lung elastic recoil are at equilibrium. The second feature is expiration, denoted as B in Figure 1A. Depending on the ventilator settings and lung condition, the gradient of the rising and falling signals can vary across patients and in the same patient over time. Therefore, the model must be able to represent these features independently. Accordingly, the gradients of inspiration and expiration of volume are features that are variable and estimable in the volume model.

The characteristic shape of the pressure waveform can vary more dramatically than the volume waveform as shown in the hand drawn Figure 1B. When pressure is a free variable, such as in VCV, the pressure waveform has several important features that convey information about lung condition and ventilator-patient interaction. Based on observation of a large number of recorded breaths, we identified five important features in the pressure waveform. Features A1 and A2 in Figure 1B determine the gradient of the inspiration. The time-varying graph of inspiration can have two distinct modes where the gradient of the signal may increase (Figure 1B, breath 1) or decrease (Figure 1B, breath 2) during inspiration. These features are hypothesized to correspond to the volume-dependent decrease in lung compliance (breath 1) or an increase in compliance due to recruitment (breath 2) (Smith et al., 2015; Hamlington et al., 2016). Note that this interpretation is only valid if the flow rate is constant during inspiration (Grasso et al., 2004).

Features B1 and B2 (Figure 1B) are related to the shape of the waveform at the start and end of the plateau pressure, which is a period of constant pressure. There may be peaks at the beginning (B1) and/or at the end (B2) of the plateau pressure, which are hypothesized to correspond to inspiratory flow resistance and patient effort, respectively (Bates, 2009; Mellema, 2013). Feature C in Figure 1B corresponds to the gradient of expiration. We also model the constant baseline pressure, known as the positive end-expiratory pressure (PEEP), because it is a key independent variable in ARDS management (Guerin, 2011; Cavalcanti et al., 2017). Note that in hybrid ventilation modes, there may be scenarios where both pressure and volume variables are partially controlled and so, in those cases, both the waveforms can be confounded in additionally complex ways and would require more nuanced interpretation.

### Constructing the Damage-Informed Lung Ventilator Model

Once we define the physiological and ventilation-related relevant features in the waveform data, we formulate the model for volume and pressure signals. During this process, we have chosen minimal number of parameters while ensuring that the model should have the ability to address the deep complexity present in the waveform data due to complex pathophysiology and patient-ventilator interactions. Additionally, the parameters have little overlap as they are not active at the same time within a given breath, and many of the parameters control only specific aspects of a given deformation such as a peak value. In this way, while we add parameters, they act locally along a breath and are tied to a deformation shape and timing that is hypothesized to be related to pathophysiology.

#### Construction of the Volume Model

Irrespective of the state variable, the models have periodic dynamics with a frequency defined by the respiratory rate (breaths/min) that should be the same in pressure and volume waveform models. In addition to this constraint, the volume model has two additional features, the rate of inspiration and expiration (A and B in Figure 1A, respectively). Volume model development begins by modeling the respiratory rate with a sinusoidal function (*f*_*s*__1_):


(1) $$f_{s\,1}=\sin\left(2\,\pi \,\theta \,t-\phi_{1}\right)-b_{1}.$$

Here, the respiratory frequency (breaths/s) is set by θ and *t* represents time in seconds while parameter ϕ_1_ allows to control the starting point in the respiratory cycle. To control the rate of inspiration or expiration while maintaining the periodicity, we create a periodic rectangular waveform function *f*_*b*__1_ by combining the sinusoidal function with hyperbolic tangent function:


(2) $$f_{b\,1}=\frac{1}{2}\,\left\{\tanh\left(a_{1}\,f_{s\,1}\right)+1\right\}.$$

To control the smoothness of the rectangular waveform, we added a smoothing parameter *a*_1_. The other terms (1/2, +1) are added to generate a rectangular waveform that has a zero-base value and unit amplitude. To control the duty cycle of the rectangular waveform that sets inspiratory:expiratory (I:E) ratio, we used parameter *b*_1_ such that the zero value of *b*_1_ corresponds to 1:1 I:E ratio. Figure 1A shows additional model features: the rate of inspiration (A) and expiration (B). To represent these rates independently, we define the volume (*V*) using the rectangular waveform as a base waveform:


(3) $$V=A_{v}\,\left(f_{v\,1}+f_{v\,2}\right),$$

where *f*_*v*__1_ term produces the inspiration part of the volume signal (feature A):


$$f_{v\,1}\,\left(i+1\right)=\left\{\frac{1}{\beta_{1}}\,f_{b\,1}\,\left(i+1\right)+\left(1-\frac{1}{\beta_{1}}\right)\,f_{v\,1}\,\left(i\right)\right\};i=1:n,$$

(4) $$f_{v\,1}=\left[f_{v\,1}\,\left(1\right)\,f_{v\,1}\,\left(2\right)\,\ldots \,f_{v\,1}\,\left(i\right)\,\ldots \,f_{v\,1}\,\left(n\right)\right]\,\frac{f_{b\,1}}{\max\left(f_{v\,1}\right)},$$

and *f*_*v*__2_ term produces the expiration part of the volume signal (feature B):


$$f_{v\,2}\,\left(i+1\right)=\left\{\frac{1}{\beta_{2}}\,f_{b\,1}\,\left(i+1\right)+\left(1-\frac{1}{\beta_{2}}\right)\,f_{v\,2}\,\left(i\right)\right\};i=1:n,$$

(5) $$f_{v\,2}=\left[f_{v\,2}\,\left(1\right)\,f_{v\,2}\,\left(2\right)\,\ldots \,f_{v\,2}\,\left(i\right)\,\ldots \,f_{v\,2}\,\left(n\right)\right]\,\frac{f_{b\,1}}{\max\left(f_{v\,2}\right)}.$$

**FIGURE 2:**
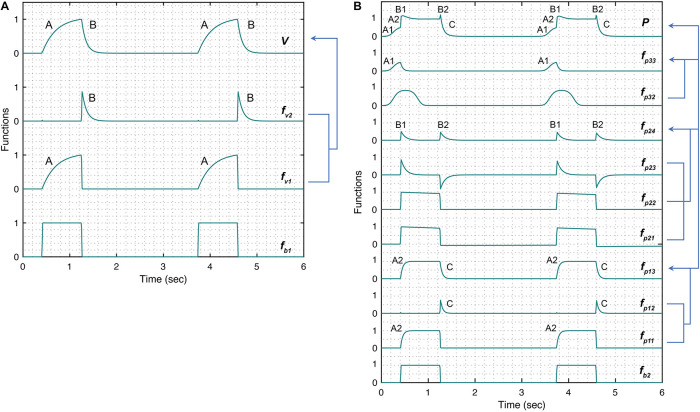
Simulated response of various terms that make up the damage-informed volume (*V*) and pressure model (*P*). **(A)** A periodic rectangular waveform *f*_*b*__1_ is used to create terms *f*_*v*__1_ and *f*_*v*__2_ through which the gradient of the rising (feature A) and falling (feature B) signals in the volume waveform are controlled, respectively. Equations 1–5 were used to simulate the response within each term with parameter values θ = 0.3, *a*_1_ = 200, *b*_1_ = 0.7, ϕ_1_ = 0, β_1_ = 30, β_2_ = 10, and *A*_*v*_ = 1. **(B)** A periodic rectangular waveform (*f*_*b*__2_) serves as a basis of the pressure model. The overall shape of the pressure waveform, which defines the gradient of the inspiration and expiration signals, is formed using *f*_*p*__13_ comprised of the rising signal of *f*_*p*__11_ (A2) and falling signal of *f*_*p*__12_ (C). The shape of the plateau pressure is defined by *f*_*p*__24_, where the output of *f*_*b*__2_ is processed via *f*_*p*__21_, *f*_*p*__22_, and *f*_*p*__23_ to produce peaks at the beginning (B1) and end (B2) of the plateau pressure. The shape of the rising signal at low volume (A1) is defined by *f*_*p*__33_, where a short pulse is produced via *f*_*p*__31_ and reshaped via *f*_*p*__32_. Note that the amplitude terms *A*_*p*__1_, *A*_*p*__2_, and *A*_*p*__3_ control the amplitude of *f*_*p*__13_, *f*_*p*__24_, and *f*_*p*__33_, respectively. Equations 6–18 were used to simulate the response of each term with parameter values θ = 0.3, *a*_2_ = 200, *b*_2_ = 0.7, ϕ_2_ = 0, *a*_3_ = 10, *b*_3_ = 0.9, ϕ_3_ = –0.6, β_3_ = β_4_ = 5, β_5_ = 1.001, β_6_ = 1.1111, *A*_*p*__1_ = 1, *A*_*p*__2_ = 0.5, *A*_*p*__3_ = 0.5, and *A*_*p*__4_ = 0. Note that the model variability shown here is independent of the ventilator mode.

Here, β_1_ and β_2_ control the gradient of the inspiration and expiration, respectively, while *A*_*v*_ controls the amplitude of the volume waveform. Figure 2A shows the volume waveform (top plot) and the constitutive terms added through with Eqs 1–5. Note that the expiration part of the breath (feature B) is generally spontaneous and could be model using a logarithmic function. We have opted for the current form of the model so that it can be converted into the ordinary differential equations-based model relatively easily in future studies where we plan to use Bayesian interference schemes (Gelman et al., 2013).

#### Construction of the Pressure Model

The pressure model has five explicit features that might be used to understand lung health and ventilator settings. These features are depicted in Figure 1B. Features A1 and A2 capture the gradient of the rising signal during inspiration at low (A1) and high (A2) volume and might be correlated with lung compliance. Features B1 and B2 capture the shape of the peaks at the beginning (B1) and end (B2) of the plateau pressure and might reflect changes in inspiratory flow resistance and patient effort, respectively. Finally, feature C captures the rate of change of the pressure during expiration. To build a model that can capture all these features and be able to estimate parameters reliably and uniquely, we used a modular approach to build the components of the pressure model where each component is controlled by set parameters that form those components.

The pressure model construction begins like the volume model, with a sinusoid. Because volume and pressure are coupled through their period (respiratory rate), we enforce this constraint by requiring that both models have the same respiratory frequency (θ) in their base periodic sinusoid:


(6) $$f_{s\,2}=\sin\left(2\,\pi \,\theta \,t-\phi_{2}\right)-b_{2}.$$

Because the pressure may lag or lead the volume depending on the ventilator mode, we include a phase shift term, ϕ_2_ in the sinusoid. To account for variations in the duty cycle of the rectangular waveform, we added the parameter *b*_2_ that defines the I:E ratio. We then create a rectangular waveform *f*_*b*__2_ as we did for the volume model using the hyperbolic tangent:


(7) $$f_{b\,2}=\frac{1}{2}\,\left\{\tanh\left(a_{2}\,f_{s\,2}\right)+1\right\}.$$

The smoothness of the rectangular waveform is controlled via the parameter *a*_2_. The five key features in pressure are represented with three additional terms: (i) *f*_*p*__13_ defines the rates of pressure change during inspiration and expiration, (ii) *f*_*p*__24_ determines the peaks at the beginning and end of the pressure plateau, and (iii) *f*_*p*__33_ specifies the gradient of the initial rising signal during inspiration, leaving us with the full the pressure model (P):


(8) $$P=f_{p\,13}+f_{p\,24}+f_{p\,33}+A_{p\,4}.$$

The constant parameter *A*_*p*__4_ corresponds to the baseline pressure value (PEEP). The rates of pressure change during inspiration and expiration (see A2 and C in Figure 1B, respectively) are:


(9) $$f_{p\,13}=A_{p\,1}\,\left(f_{p\,11}+f_{p\,12}\right),$$

where *f*_*p*__11_ term produces the rising part of the pressure signal:


$$f_{p\,11}\,\left(i+1\right)=\left\{\frac{1}{\beta_{3}}\,f_{b\,2}\,\left(i+1\right)+\left(1-\frac{1}{\beta_{3}}\right)\,f_{p\,11}\,\left(i\right)\right\};i=1:n,$$

(10) $$f_{p\,11}=\left[f_{p\,11}\,\left(1\right)\,f_{p\,11}\,\left(2\right)\,\ldots \,f_{p\,11}\,\left(i\right)\,\ldots \,f_{p\,11}\,\left(n\right)\right]\,\frac{f_{b\,2}}{\max\left(f_{p\,11}\right)},$$

and *f*_*p*__12_ term produces the falling part of the pressure signal:


$$f_{p\,12}\,\left(i+1\right)=\left\{\frac{1}{\beta_{4}}\,f_{b\,2}\,\left(i+1\right)+\left(1-\frac{1}{\beta_{4}}\right)\,f_{p\,12}\,\left(i\right)\right\};i=1:n,$$

(11) $$f_{p\,12}=\left[f_{p\,12}\,\left(1\right)\,f_{p\,12}\,\left(2\right)\,\ldots \,f_{p\,12}\,\left(i\right)\,\ldots \,f_{p\,12}\,\left(n\right)\right]\,\frac{f_{b\,2}}{\max\left(f_{p\,12}\right)}.$$

Here, β_3_ and β_4_ control the gradient during inspiration and expiration, respectively. The next set of features, the peaks at the beginning and end of plateau pressure (B1 and B2 in Figure 1B), are represented by:


(12) $$f_{p\,24}=A_{p\,2}\,\frac{f_{p\,23}}{\max\left(f_{p\,23}\right)},$$

where *f*_*p*__21_ and *f*_*p*__22_ terms create the initial shape of peaks at the plateau pressure:


$$f_{p\,21}\,\left(i+1\right)=\frac{1}{\beta_{5}}\,\left\{f_{p\,21}\,\left(i\right)+\left\{f_{b\,2}\,\left(i+1\right)-f_{b\,2}\,\left(i\right)\right\}\right\};i=1:n,$$

(13) $$f_{p\,21}=\left[f_{p\,21}\,\left(1\right)\,f_{p\,21}\,\left(2\right)\,\ldots \,f_{p\,21}\,\left(i\right)\,\ldots \,f_{p\,21}\,\left(n\right)\right],$$

(14) $$f_{p\,22}=f_{p\,21}\,f_{b\,2},$$

and *f*_*p*__23_ term further reshapes both the peaks:


$$f_{p\,23}\,\left(i+1\right)=\frac{1}{\beta_{6}}\,\left\{f_{p\,23}\,\left(i\right)+\left\{f_{p\,22}\,\left(i+1\right)-f_{p\,22}\,\left(i\right)\right\}\right\};i=1:n,$$

(15) $$f_{p\,23}=a\,b\,s\,\left(\left[f_{p\,23}\,\left(1\right)\,f_{p\,23}\,\left(2\right)\,\ldots \,f_{p\,23}\,\left(i\right)\,\ldots \,f_{p\,23}\,\left(n\right)\right]\right).$$

The parameters β_5_ and β_6_ control the shape of both the peaks, which are present at the plateau pressure. Finally, the gradient of the initial rate of inspiration (Figure 1B, A1) is modeled by:


(16) $$f_{p\,33}=A_{p\,3}\,\frac{f_{p\,32}\,\left\{1-\left(f_{p\,11}+f_{p\,12}\right)\right\}}{\max\left[f_{p\,32}\,\left\{1-\left(f_{p\,11}+f_{p\,12}\right)\right\}\right]},$$

where a short pulse is produced via *f*_*p*__31_:


(17) $$f_{p\,31}=\sin\left(2\,\pi \,\theta \,t-\phi_{3}\right)-b_{3},$$

and reshaped via *f*_*p*__32_ term:


(18) $$f_{p\,32}=\frac{1}{2}\,\left\{\tanh\left(a_{3}\,f_{p\,31}\right)+1\right\}.$$

The position, shape and gradient of the rising signal, produced by *f*_*p*__33_ term are controlled using the parameters ϕ_3_, *b*_3_ and *a*_3_, respectively. Figure 2B shows the pressure waveform and the constitutive terms added through Eqs 6–18. The pressure waveform (top plot) composed of the three terms *f*_*p*__13_,*f_*p*_*_24_, and *f*_*p*__33_ that capture the gradient of the inspiration (A2) and expiration signals (C), the shape of the plateau pressure (B1 and B2), and the shape of the rising signal at low volume (A1), respectively.

### Mouse Mechanical Ventilation Experiments

Three 8 to 10-week-old female BALB/c mice (Jackson Laboratories, Bar Harbor, ME, United States) were studied under University of Colorado, Anschutz Medical Campus Institutional Animal Care and used Committee (IACUC)-approved protocol (#00230). The mice weighed 18.6, 19.1, and 19.9 g. Anesthesia was induced with an intraperitoneal (IP) injection of 100 mg/kg Ketamine and 16 mg/kg Xylazine, a tracheostomy was performed with a blunted 18 ga metal cannula, and ventilation was started on the flexiVent small animal ventilator (SCIREQ, Montreal, QC, Canada). Anesthesia was maintained with 50 mg/kg Ketamine or 50 mg/kg Ketamine with 8 mg/kg Xylazine at 30 min intervals along with 50 μL IP 5% dextrose lactated Ringer’s solution. Heart rate was monitored via electrocardiogram.

Baseline ventilation, consisting of a tidal volume (Vt) = 6 ml/kg, PEEP = 3 cmH_2_O, and respiratory rate (RR) = 250 breaths/min, was applied for a 10 min stabilization period with recruitment maneuvers at 3 min intervals. Pressure and volume were recorded with a custom flowmeter based on our previously published design (Jawde et al., 2018). Four types of ventilation were recorded for analysis: (1) VCV-PEEP0, consisting the baseline ventilation with PEEP = 0 cmH_2_O, (2) VCV-PEEP12 that was the baseline ventilation with PEEP = 12 cmH_2_O, (3) HighPressure-PEEP0 that consisted of a inspiratory pressure (Pplat) = 35 cmH_2_O and PEEP = 0 cmH_2_O with RR = 60 breaths/min, and (4) PCV-PEEP0 with Pplat = 10 and PEEP = 0 cmH_2_O with RR = 70 breaths/min. After the initial measurements of the healthy lung, lung injury was induced with a 0.15 ml lavage with warm saline (Mellenthin et al., 2019). This fluid was pushed into the lung with an additional 0.3 ml air, and suction was applied to the tracheal cannula with an approximate return of 0.05 ml. The mouse was then ventilated for 10 mins with the HighPressure-PEEP0 settings. The sequence of four measurement ventilation patterns (above) was repeated, then the mouse received 0.8 mg/kg IP pancuronium bromide to suppress respiratory efforts, and the measurements were repeated again.

### Human Data Collection

Between June 2014 and January 2017, 140 adult patients admitted to the University of Colorado Hospital medical intensive care unit (MICU) at risk for or with ARDS and requiring mechanical ventilation were enrolled within 12 h of intubation (Wheeler and Bernard, 2007). At risk patients were defined as intubated patients with hypoxemia and a mechanism of lung injury known to cause ARDS, who had not yet met chest X-ray or oxygenation criteria for ARDS. To facilitate the capture of continuous ventilator data, only patients ventilated with a Hamilton G5 ventilator were included. Patients requiring mechanical ventilation only for asthma, COPD, heart failure, or airway protection were excluded. Additionally, patients less than 18 years of age, pregnant, or imprisoned were excluded. The University of Colorado Hospital utilizes a ventilator protocol that incorporated the ARDS network low tidal volume protocol with the low PEEP titration table. The Colorado Multiple Institutional Review Board approved this study and waived the need for informed consent.

Baseline patient information including age, gender, height, and initial P/F ratio were collected. Human patient data shown in Figures 6A–C belong to a 62 years old female with an initial P/F 70, height 165 cm, and weight 127 kg. The data shown in Figures 6D–F belongs to a 47 years old male, initial P/F 230, height 177 cm, and weight 96.9 kg. Continuous ventilator data were collected using a laptop connected to the ventilator and using Hamilton Data Logger software (Hamilton, v5.0, 2011) to obtain pressure, flow, and volume measurements. Additionally, the DataLogger software allowed collection of ventilator mode and ventilator settings based on mode [i.e.: set tidal, respiratory rate, PEEP, and fraction inspired oxygen (FiO_2_)]. Data were collected until extubation or for up to 7 days per patient.

### Parameter Estimation Methodology

The damage-informed lung ventilator model is a complex model and we estimate its parameters for mouse and human clinical ventilator data. In clinical situations, the patient data are variable and often nonstationary because of interventions, patient-ventilator interactions, changes in health, etc., leading to complex parameter estimation issues. Moreover, the model we develop here is not likely to be structurally identifiable (Westwick and Kearney, 2003; Schoukens et al., 2016; Albers et al., 2019c). However, formally computing identifiability properties here is subtle because many parameters in the model functionally affect only part of the breath. This feature helps facilitate the convergence of parameter estimates and potentially leads to the uniqueness of those estimates, although because the DILV model is neither linear nor convex, there is no guarantee of unique global optima and no way of guaranteeing that the optimal solution we compute is a, or the, global optimum. Nevertheless, this feature—parameters being active at different times during a breath—also makes formal structural identifiability calculations complex to compute. These complexities force us to address four issues, (1) computational estimation methodology, (2) management of parameter identifiability issues and parameter selection methods, (3) uncertainty quantification, and (4) estimation evaluation methodology.

#### Computational Estimation Methodology

Our needs require an estimation methodology that allows us to estimate states and parameters of the model effectively and the respective uncertainties in the estimated parameters. While stochastic methods, e.g., Markov Chain Monte Carlo (MCMC) (Gelman et al., 2013), might guarantee to find global minima and quantifying uncertainty in the estimated parameters values, they are generally quite slow. On the other hand, deterministic methods, e.g., Nelder-Mead optimization (Nelder and Mead, 1965), are substantially faster and by choosing many initial conditions, we are still able to quantify the uncertainty of a solution. In particular, here we infer parameters with a standard class of deterministic, multivariate, constrained nonlinear optimization methods, interior-point methods (Bertsimas and Tsitsiklis, 1997; Nocedal, 2006), a choice that is not critical among constrained, nonlinear optimization algorithms. As such, we focus on a smoothing task that employs deterministic nonlinear optimization methods that work well with careful parameter selection and constraints and can be used to quantify uncertainty.

#### Management of Parameter Identifiability and Parameter Selection Methods

Irrespective of the estimation methodology, identifiability failure—non-uniqueness of non-convergence of solutions—can occur. In particular, the DILV model is likely not identifiable. To mitigate this problem, we use three different approaches to minimize impacts of identifiability failures while estimating parameters for a given waveform data. First, the model was constructed such that each parameter in the model contributes to the specific feature in the volume and pressure curves, allowing the parameter to be estimated relative to the specific, time-limited feature by definition. Second, we constrain the ranges of all parameters to lie within physically possible values. And third, we do not estimate every parameter in all circumstances but rather limit parameters estimated to those relevant for a given setting and fix many low-impact, low-sensitivity parameters (Law et al., 2015; Asch et al., 2016; Albers et al., 2019b).

In more detail, the DILV model includes two state variables, volume and pressure with one overlapping parameter, the frequency of the breath (θ). The volume model has six parameters (*a*_1_, *b*_1_, ϕ_1_, β_1_, β_2_, and *A*_*v*_). The pressure model has fourteen parameters (*a*_2_, *b*_2_, ϕ_2_, *a*_3_, *b*_3_, ϕ_3_, β_3_, β_4_, β_5_, β_6_, *A*_*p*__1_, *A*_*p*__2_, *A*_*p*__3_, and *A*_*p*__4_). Many of these parameters can effectively be uniquely estimated because they operate on a particular part of the waveform, e.g., parameters that control the gradient of the rising signal (β_1_, β_3_) and falling signal (β_2_, β_4_) and amplitudes of the waveforms (*A*_*v*_, *A*_*p*__1_). Nevertheless, there are parameters that are not necessarily uniquely estimable, e.g., parameters that control feature A1 (*a*_3_, *b*_3_, ϕ_3_, and *A*_*p*__3_) and features B1 and B2 (β_5_, β_6_, and *A*_*p*__2_).

#### Uncertainty Quantification

Because we use deterministic optimization methods whose final solution depends on the initialization, we quantify uncertainty by randomly sampling a set optimization initialization for the parameters we estimate from a uniform distribution within a bounded interval (upper and lower bounds) centered around initial values (Smith, 2013; Albers et al., 2019a). The boundaries of the intervals were chosen to exclude parameter variation that was unrealistic. The upper and lower boundaries of the intervals were chosen by computing parameters that provide a qualitative agreement between the model and the measured response. The optimal parameter estimate is then represented as a probability density in a similar way as is created using MCMC, allowing us to understand how informative, unique, and uncertain a given parameter solution set is. Additionally, we have uncertainty for individual breaths—we estimate every breath many times computing an uncertainty in by-breath parameter estimation—and uncertainty due to variation in many breaths over time. This allows us to both resolve and quantify single-breath features, and how those features vary over time, for different breaths, and even between individuals.

#### Estimation Evaluation Methodology

The output of this computational method is a *distribution* of optimal solutions. Through this distribution, we understand the robustness of the solution and the uncertainty of the solution. If the distribution of solutions has multiple modes with similar error then we can conclude that there are multiple plausible solutions. Similarly, if the distribution of solutions is narrow or wide with similar errors, we can conclude that the model either does or does not depend highly on a given parameter. And finally, it is the distribution of parameter solutions that *define the phenotype computed by the model* in the sense that the distribution of parameters explains the by-breath characterization of the patient. We verify a model’s ability to represent data by computing the mean squared error between the model computed with parameter values taken as the medians of the optimally computed solution and the data. There is uncertainty in these MSE values too, and if one model has a lower MSE value than another with non-overlapping uncertainty in MSE, we conclude that the model with the lower median MSE more accurately represents the data.

## Results

In this section, we qualitative and quantitative validate the DILV model using numerical simulations and measured data, respectively.

### Qualitative Model Validation, Parametric Descriptions, and Simulated Model Variability

The first step in model validation is the qualitative validation (Jolliffe and Stephenson, 2012) that involves demonstrating the model has enough flexibility to recapitulate the key features that are often seen in clinically collected volume and pressure waveform data. We then identify the parameters that correspond to hypothesized lung physiology by analyzing simulated pressure and volume waveforms.

#### Volume Model Flexibility

**FIGURE 3:**
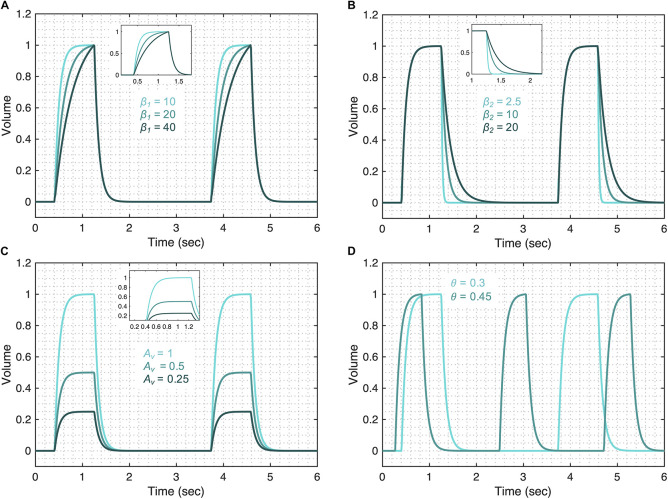
Demonstrating the volume model flexibility by varying parameters that alter characteristic features of the volume waveform. The gradient of the rising and falling signals can be altered using the **(A)** β_1_ and **(B)** β_2_ parameters, respectively. Increased values of these parameters increase the transient time for the signal to reach the same volume level. **(C)** The amplitude of the waveform can be altered using the parameter *A*_*v*_. **(D)** Changes in the respiratory frequency (θ) change the period of the breath. The output of the model (*V*) was calculated using Eqs 1–5 while considering θ = 0.3, *a*_1_ = 200, *b*_1_ = 0.7, ϕ_1_ = 0, β_1_ = 30, β_2_ = 10, and *A*_*v*_ = 1. The respective variation in the functions that make the volume model is shown in Supplementary Figure 1 for each case. Note that the model variability shown here is independent of the ventilator mode.

The volume model flexibility is demonstrated in Figure 3 where we vary the rates of inspiration (feature A) and expiration (feature B) through the terms *f*_*v*__1_ and *f*_*v*__2_, which are controlled by parameters β_1_ and β_2_ (Figures 3A,B), respectively. The full variability of terms *f*_*v*__1_ and *f*_*v*__2_ is shown in Supplementary Figure 1. Additionally, the amplitude of the volume waveform is controlled by *A*_*v*_ (Figure 3C), variations in respiratory rate are controlled by θ (Figure 3D). Finally, the I:E ratio, the starting point of the breath in the breathing cycle and the smoothness of the waveform are set by *b*_1_, ϕ_1_, and *a*_1_ as shown in Supplementary Figure 2, respectively.

#### Pressure Model Flexibility

**FIGURE 4:**
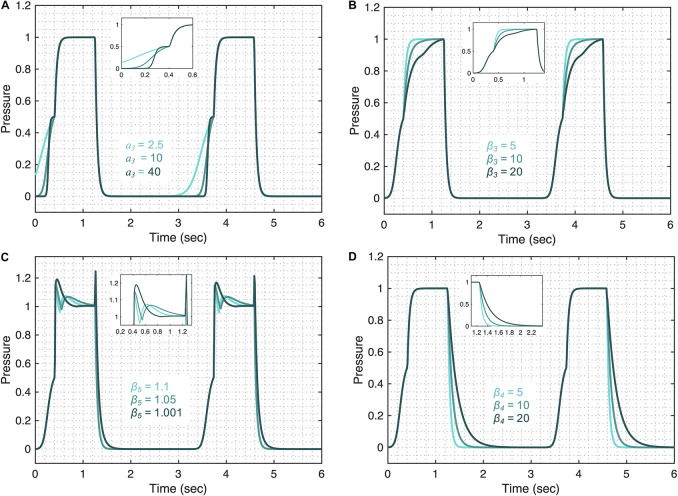
Demonstrating the pressure model flexibility by altering relevant features in the pressure waveform. The initial gradient of the pressure signal during inspiration at low volume (feature A1) is controlled by **(A)** the *a*_3_ parameter. **(B)** The gradient of the rising signal after the inflection point (feature A2), is controlled by the β_3_ parameter. **(C)** The shapes of the peaks at the beginning (feature B1) and at the end (feature B2) of the plateau are regulated by the β_5_ parameter when *A*_*p*__4_ = 0.5. **(D)** The gradient of the falling signal (feature C) during expiration can be modified by the β_4_ parameter. Equations 6–18 were used to simulate the response of the pressure model while considering θ = 0.3, *a*_2_ = 200, *b*_2_ = 0.7, ϕ_2_ = 0, *a*_3_ = 10, *b*_3_ = 0.9, ϕ_3_ = –0.6, β_3_ = β_4_ = 5, β_5_ = 1.001, β_6_ = 1.1111, *A*_*p*__1_ = 1, *A*_*p*__2_ = 0.5, *A*_*p*__3_ = 0.5, and *A*_*p*__4_ = 0. The respective variations in the terms that make the pressure model are shown in the Supplementary Figure 3 for each case. Note that the model variability shown here is independent of the ventilator mode.

The pressure model flexibility is demonstrated in Figure 4 where we vary the five features of the pressure waveform via respective parameters: variation in the rate of change of the pressure before (A1 in Figure 1B) and after (A2 in Figure 1B) the inflection point during inspiration; the shape of the peaks at the beginning (B1 in Figure 1B) and end (B2 in Figure 1B) of the plateau pressure; and variation in the rate of change of the pressure during expiration (C in Figure 1B). In brief, these features are controlled by the following parameters.

The initial gradient of the pressure during inspiration (A1) is controlled by the *a*_3_ parameter such that higher values of *a*_3_ result in a slower rising signal (Figure 4A). The full variation that these terms are capable of is shown in Supplementary Figure 3. The shape of the initial gradient signal (A1) before the inflection point can be altered using the *b*_3_ parameter (Supplementary Figure 4A) and the amplitude of the initial gradient alteration is controlled by the *A*_*p*__3_ parameter (Supplementary Figure 4B). By setting *A*_*p*__3_ parameter to zero, feature A1 can be removed from the pressure waveforms. The rate of inspiratory pressure after the inflection point (A2) is specified by β_3_ such that higher values of β_3_ result in a slower rising signal (Figure 4B). The shapes of the peaks at the beginning (B1) and end (B2) of the plateau pressure are controlled by several parameters. The overall shape of the peaks is controlled by the β_5_ (Figure 4C) and the sharpness of these peaks can be further altered by β_6_ (Supplementary Figure 4C). The amplitude of the peaks is controlled by *A*_*p*__2_ (Supplementary Figure 4D). By setting *A*_*p*__2_ parameter to zero, features B1 and B2 can be turned off. Additional control of features B1 and B2 can be achieved in combination with parameter β_3_ shown in Supplementary Figures 4E,F. The rate of pressure decrease during expiration (C) is specified by β_4_ such that higher values of β_4_ result in a slower falling signal (Figure 4D). Finally, the amplitude of the plateau pressure can be altered using the *A*_*p*__1_ parameter (Supplementary Figure 4G). The I:E ratio is defined by the *b*_2_ parameter in the same way that parameter *b*_1_ controls the I:E ratio in the volume model (Supplementary Figure 2A). A summary of model parameters is provided in Table 1.

**TABLE 1:** Interpreting damaged-informed lung ventilator model parameters.

**Parameter**	**Model relevance**	**Physiological relevance (with increased values)**
**Volume model**		
θ	Number of breaths/s. Higher values result in a higher number of breaths/s	–
*a*_1_	Smoothness of the square waveform (*f*_*b*__1_). Higher values result in a sharper transition	–
*b*_1_	I:E ratio. Higher values result in smaller inspiration cycle	–
Φ_1_	Starting of the inspiration point. Higher value results in a more delayed response	–
**β_1_**	Gradient of the rising signal. Higher values result in a slower rising signal	Lower compliance and/or higher resistance
**β_2_**	Gradient of the falling signal. Higher values result in a slower falling signal	Higher expiration time constant
***A*_*v*_**	Peak amplitude	Higher overall compliance
**Pressure model**		
*b*_2_	I:E ratio. Higher values result in a smaller inspiration cycle	–
*a*_2_	Smoothness of the square waveform. Higher values result in a sharper transition	–
Φ_2_	Starting of the *f*_*b*__2_ function. Higher value results in a more delayed response	–
***a*_3_, *b*_3_**	Gradient of the pressure signal at low volume. Higher values result in a slower rise	Higher low volume compliance
Φ_3_	Starting of the inspiration point. Higher value results in a more delayed response	–
**β_3_**	Gradient of the rising signal after inflection point. Higher values result in a slower rising signal	Higher high-volume compliance
β_4_	Gradient of the falling signal. Higher values result in a slower falling signal	–
β_5_, β_6_	Shape of plateau. Higher value means a sharper peak	Associated with ventilator desynchrony
***A*_*p*__1_**	Peak amplitude	Lower high-volume compliance
*A*_*p*__2_	Amplitude of the peaks at the plateau	
***A*_*p*__3_**	Higher values increase the amplitude of *f*_*p*__33_ function	Moves upper inflection point up
*A*_*p*__4_	Pressure base line value	PEEP

*The parameters that are correlated with known measures of lung physiology are in bold.*

### Qualitatively Relating the Model Parameters With the Lung Function Parameters

In order to be able to use the DILV model parameters to infer lung health, it is required that we set up the initial framework to correlate the model parameter with the lung condition, given the model parameters are not physiological parameters but rather chosen to control specific features in the waveform data. On this account, the DILV model is anchored to physiology through variations or deviations from nominal breath waveforms that are hypothesized to relate to lung conditions observed in mechanical ventilation data collected in lab and clinical settings. Throughout the manuscript, we list the proposed physiological interpretations of the parameters – a short description of how the model parameters contribute to the model is provided in Table 1 – but here we will go into interpretative depth regarding the qualitative correlation between model parameters and the fundamental characteristics of the lung such as compliance and resistance. In this qualitative interpretation, we consider only one variable (volume in PCV and pressure in VCV) while assuming the other waveform does not change breath-to-breath (pressure in PCV and volume in VCV).

We first show how the changes in the volume model parameters can be qualitatively related to changes in lung compliance and resistance when the volume variable is free. For that, we focus on three model parameters that might have direct physiological meaning: β_1_, β_2_, and *A*_*v*_. The first parameter, β_1_ might be inversely correlated with lung compliance as higher values of β_1_ result in a lower inspiratory flow rate (Figure 3A and Supplementary Figure 5A). During PCV, the inspiratory flow rate will decrease with reduced compliance or increased resistance. A second parameter, β_2_, controls the gradient of expiration and is captured as feature B in Figure 1A. Higher values of β_2_ result in a longer expiration (cf. Figure 3B and Supplementary Figure 5A) and so β_2_ is directly proportional to the expiratory time constant, which is the product of resistance and compliance. Finally, parameter *A*_*v*_ controls the amplitude of the volume waveform and for the same pressure waveform (in PCV) indicates a direct correlation with compliance (Figure 3C and Supplementary Figure 5A). In VCV, parameter *A*_*v*_ would present the tidal volume, which is set by the ventilator.

The pressure model has five parameters that may reflect aspects of lung compliance during VCV: *a*_3_, b_3_, β_3_, *A*_*p*__1_, and *A*_*p*__3_. The parameter *a*_3_, which controls feature A1 (Figure 4A), may be directly correlated with low-volume compliance as higher values of *a*_3_ result in slower pressure rise at low volume while maintaining the shape of the gradient (Supplementary Figure 5B). Additionally, parameter *b*_3_ can also be used to control feature A1 (Supplementary Figure 5B) and is directly related to the low-volume compliance. A third parameter, β_3_ controls the rate of pressure increase above the inspiratory inflection point (A2), and higher values of β_3_ result in slower pressure increase (Supplementary Figure 5B), indicating β_3_ might be correlated with high-volume compliance during VCV. A fourth parameter, *A*_*p*__1_, defines the plateau pressure with higher values of *A*_*p*__1_ yielding higher plateau pressures (Supplementary Figure 5B), indicating an inverse correlation between *A*_*p*__1_ and compliance during VCV. Finally, change in the upper inflection point (UIP) can be directly related to the *A*_*p*__3_ parameter such that higher values of *A*_*p*__3_ increase the UIP pressure as shown in Supplementary Figure 5B. During PCV, these (and other) parameters may be directly controlled via a ventilator.

It is important to note that these interpretations are qualitative and valid only when a change is observed in one of the variables (volume or pressure) while the other waveform (pressure or volume) is held fixed. In cases where both the volume and pressure waveforms change simultaneously, additional interpretation is needed to establish the relationships between pressure and volume parameters. For example, in the pressure signal, interpretation of feature A2 with respect to A1 will be valid only during the constant flow signal (Grasso et al., 2004). Similarly, when there is a change in the amplitude of volume and pressure simultaneously, we use the *A*_*v*_/*A*_*p*__1_ to assess compliance.

### Damage-Informed Lung Ventilator Model Quantitative Verification for Experimental Mouse Model Ventilator Data

To demonstrate the effectiveness of the DILV model, we now quantitatively validate the model by estimating parameters for data sets corresponding to different phenotypes—e.g., injured versus healthy. We then show that differences in the estimated parameter values reflect different phenotypic states in a manner that is consistent with expected changes due to acute lung injury. Here we consider data from PCV and VCV, in healthy and lung-injured mice, and in the absence and presence of VD.

#### In Pressure Controlled Ventilation, the Model Outcomes Align With the Injury Status and Single Compartment Model

**FIGURE 5:**
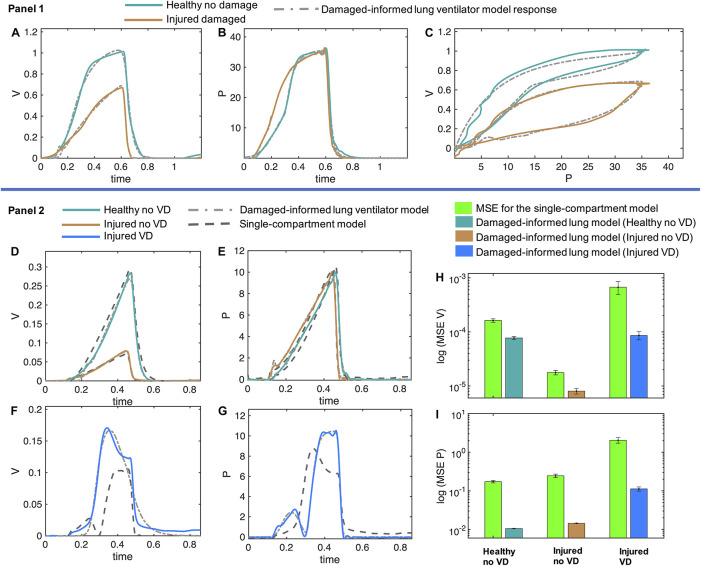
Volume and pressure models’ responses closely agree with the experimental data from the representative mice in healthy and injured condition. **(A–C)** In Panel 1, the measured breaths of first mouse are shown in healthy and injured conditions (solid lines) while the damaged-informed lung ventilator (DILV) model response is shown in dashed lines. Equations 1–18 were used to generate the best-fit model response using estimated mean parameter values shown in Table 1, respectively. **(D–G)** In Panel 2, three representative breaths of mouse two is shown here, which are corresponding to three different lung conditions (solid lines) while the damaged-informed lung ventilator model response is shown in the dashed-dot lines. For these breaths, histograms of the initial guesses and estimated parameters are shown in Supplementary Figure 6A. The response of the single-compartment model is shown in dashed-dashed lines. **(F–I)** The cumulative values of mean squares errors (MSE) of the two models are compared here. These values corresponded to sixty breaths for healthy no ventilator dyssynchrony (VD) and injured no VD cases while ten breaths for injured VD case. For each case and breath, the estimated parameter values and MSE are shown in the Supplementary Figures 6B–D. All the data shown here was collected in PCV (see “Materials and Methods”).

Figures 5A,B, panel 1 shows two different pressure-controlled breaths recorded in a healthy mouse (green) and after a lung injury induced by injurious lavage and mechanical ventilation (orange). The pressure-volume loops (Figure 5C) show a reduction in lung compliance and an increase in hysteresis that is characteristic of acute lung injury. The DILV model estimated states (dashed-dot lines) show the same trends. The estimated parameter values and the respective uncertainty for individual breaths are shown in Table 2 with bold indicating physiological relevance.

**TABLE 2:** Estimated model parameters obtained from the optimization scheme for the results shown in Figure 5 (Panel 1) and Figure 6 (human patient 1) correspond to the mouse and human data, respectively.

	**Figure 5 (Panel 1)**		**Figure 6 (Patient 1)**	
**Parameters**	**Healthy**	**Injured**	**Beginning**	**Ending**
θ	0.66 ± 0.000	0.66 ± 0.000	0.34 ± 0.000	0.35 ± 0.000
*a*_1_	9.67 ± 0.385	15.41 ± 0.397	7.51 ± 0.258	7.57 ± 0.282
*b*_1_	0.59 ± 0.006	0.53 ± 0.006	0.66 ± 0.000	0.63 ± 0.000
Φ_1_	0.09 ± 0.007	–0.01 ± 0.007	0.42 ± 0.003	0.51 ± 0.002
**β_1_**	**112.97 ± 9.733**	**458.95 ± 8.179**	**10.96 ± 0.223**	**12.53 ± 0.240**
**β_2_**	**28.35 ± 10.490**	**10.60 ± 10.590**	**12.42 ± 0.051**	**12.64 ± 0.039**
***A*_*v*_**	**1.03 ± 0.006**	**0.70 ± 0.003**	**402.61 ± 0.368**	**337.08 ± 0.531**
*a*_2_	52.06 ± 1.440	32.11 ± 1.514	27.39 ± 0.118	17.73 ± 0.163
*b*_2_	0.80 ± 0.002	0.66 ± 0.001	0.67 ± 0.000	0.66 ± 0.000
Φ_2_	0.34 ± 0.002	0.09 ± 0.002	0.35 ± 0.002	0.32 ± 0.001
***a*_3_**	**2.55 ± 0.334**	**5.11 ± 0.205**	**7.71 ± 0.183**	**18.37 ± 0.146**
***b*_3_**	**1.00 ± 0.002**	**0.53 ± 0.003**	**0.91 ± 0.000**	**0.90 ± 0.000**
Φ_3_	0.00 ± 0.002	0.00 ± 0.005	0.15 ± 0.001	0.14 ± 0.001
**β_3_**	**50.00 ± 0.388**	**100.84 ± 0.262**	**7.25 ± 0.157**	**12.88 ± 0.147**
β_4_	18.54 ± 0.239	11.93 ± 0.193	1.95 ± 0.121	2.52 ± 0.064
β_5_	–	–	1.0014 ± 0.0001	1.0048 ± 0.0001
β_6_	–	–	1.1262 ± 0.0012	1.0771 ± 0.0055
***A*_*p*__1_**	**35.53 ± 0.018**	**35.02 ± 0.017**	**16.58 ± 0.018**	**11.64 ± 0.008**
*A*_*p*__2_	–	–	3.80 ± 0.011	2.48 ± 0.022
***A*_*p*__3_**	**14.00 ± 0.081**	**9.60 ± 0.077**	**3.90 ± 0.022**	**4.10 ± 0.016**
*A*_*p*__4_	0.06 ± 0.035	0.00 ± 0.021	19.98 ± 0.011	17.91 ± 0.004
*Cs*	0.030	0.019	26.80	31.40
*Cd*	0.029	0.020	24.28	28.96

*The error values were determined using the standard error of the mean for individual breaths. To quantify uncertainty in parameter estimates, each individual breath was estimated 1,000 times. The parameters that are correlated with a known measure of lung physiology are in bold. Here, *Cs* and *Cd* are lung compliance values extracted by fitting the single-compartment model to data (*Cs*) and from the damaged-informed lung ventilator model (*Cd*) = *A_*v*_/A_*p*_*_1_.*

In the volume model, the injured lung showed a lower inspiratory flow rate, quantified by an increase in β_1_, and a faster expiration, quantified by a reduction in both β_2_ and *A*_*v*_ than the healthy lung model estimates. Given that the inspiratory pressures remain unchanged, this suggests a reduction in lung compliance, and the associated decrease in the expiratory time constant (See Table 1).

Physiologic interpretation of the pressure model is limited because of the use of PCV. In this case, the pressure signal is prescribed by the piston ventilator and the observed differences between the healthy and injured lungs are a result of the ventilator control system algorithms. Hence, the respective changes in the parameters’ values, such as an increase in parameters *a*_3_ and β_3_ correspond to the changes in the ventilator dynamics and not in the respiratory mechanics. These results make an important point: it is essential to see the relative change in the parameters that control these features and to synthesize the model-based inference in a holistic fashion, instead of focusing on any one parameter or feature in isolation.

To analyze changes in lung mechanics in a manner that accounts for ventilator settings, we define the lung compliance *Cd* = *A*_*v*_/*A*_*p*__1_ (Table 2), which is the ratio of volume and pressure model amplitudes. As expected, *Cd* decreases with injury. Furthermore, *Cd* shows a strong correlation with compliance (*Cs*) calculated with the single-compartment model (Table 2; Mellema, 2013).

#### Damaged-Informed Lung Ventilator Model Accurately Captures Mouse Model Data With Ventilator Dyssynchrony While the Single-Compartment Model Is Unable to Capture This Variability

In the previous section, we demonstrate that the DILV model can accurately estimate a single breath. We did not, however, validate that the model has enough flexibility to account for large variations in the waveform data, or that the DILV model can estimate a large number of breaths reliably while maintaining unique and consistent solutions of parameters values for each breath. To show these characteristics, we estimate a large number of breaths for the second mouse in three different lung conditions: (1) healthy breaths without VD, (2) injured breaths without VD, and (3) injured breaths with VD (See “Materials and Methods”). In the 1st and 2nd cases, we selected sixty breaths in a sequence from the random location out of 424 and 128 breaths, respectively. In the 3rd case, we manually selected ten breaths out of 332 breaths that had VD from the data set containing breaths with and without VD. Here, we define VD as any substantial respiratory effort because the flexiVent small animal ventilators do not allow the subject to trigger a breath. A representative breath for each case is shown in Figures 5D–G, panel 2 along with the DILV model response at the optimum parameter values, which are listed in Supplementary Table 1. Histograms of the initial guesses and estimated parameters are shown in the Supplementary Figure 6A. We observed minor variability in most of the estimated parameters values for individual breaths, suggesting that those features are modularly controlled by the respective parameter. We do observe high variability in some parameters (*a*_1_, β_1_,*a*_3_, and β_3_) due to the low sensitivity of the model for those parameters (Supplementary Figure 6A).

To demonstrate what is gained by the DILV model we compare it to single-compartment model (Bates, 2009) estimated resistance and compliance to estimate the same breaths for each case (Figure 5, panel 2). The single-compartment model has substantially larger estimated mean squares errors (MSE) and these errors increase as the mouse lung condition worsens and in the presence of VD (Figures 5H,I and Supplementary Figures 6B–D). Consequently, in terms of lung compliance values, the two models’ outcomes closely agree in the healthy lung case but then diverge somewhat for the injured lung and more substantially during VD (Supplementary Figures 6E–G). These discrepancies have their root in the limited ability of the single-compartment model to estimate VD (Figures 5F,G), errors that are quantified by the MSE between model estimates and data (Supplementary Figures 6B–D). Note that the calculated compliance values include the effects of muscle effort, which explain the differences in compliance with and without VD in the same injured mouse.

#### In Volume-Controlled Ventilation, the Pressure Model Outcomes Align With the Injury Status and the Single Compartment Model

Above we verified the DILV model using data sets collected during PCV, where the estimated volume model parameters reflected the lung dynamics since the volume was the free variable. We now consider data collected during VCV so that the estimated pressure model parameters reflect changes in lung condition. Here, we consider variations in PEEP during low tidal volume ventilation (VCV). The pressure model indicates a reduction in compliance in the injured lung as quantified by lower values of parameters *a*_3_, *b*_3_, and β_3_, and elevated estimates of in *A*_*p*__1_ (Table 1, Supplementary Figure 7, and Supplementary Table 1). In contrast to the PCV results shown in Figure 5, differences in parameter estimates between healthy and injured lungs in the pressure model were much larger compared to those estimated differences in the volume model. This is expected since the tidal volumes were approximately equal during VCV, and the reduction in compliance is reflected in increased pressure. This effect can be inferred by analyzing the *A*_*v*_/*A*_*p*__1_ ratio showing a reduction in the injured cases at both the PEEP settings (Supplementary Table 1). We also note that the healthy lung becomes stiffer at PEEP = 12 cmH_2_O due to strain stiffening. In contrast, the injured lung becomes more compliant at high PEEP, which our previous studies in this injury model attribute to recruitment (Mellenthin et al., 2019).

### Damage-Informed Lung Ventilator Model Quantitative Verification for Intensive Care Unit Patient-Ventilator Data

The DILV model is intended to be used with both laboratory data and clinical ventilator data where standard models, such as the single-compartment model, cannot recapitulate all of the potentially relevant waveform features. To show the clinical applicability of the DILV model we consider waveform data of two ICU patients, the first—patient 1—includes waveform data without VD and the second—patient 2—has waveform data with VD. For each case, we estimate each individual breath 1,000 times to quantify uncertainty in parameter estimates for each of 263 breaths to quantify uncertainty in parameter estimates across many breaths. These data were recorded near extubation when ARDS had nearly resolved. For both patients, the ventilator was operating in patient-triggered mode, a ventilator mode that is not possible in our mouse ventilators but is commonly used in the ICU.

#### Intensive Care Unit Data-Driven Verification in the Absence of Ventilator Dyssynchrony (Patient 1)

**FIGURE 6:**
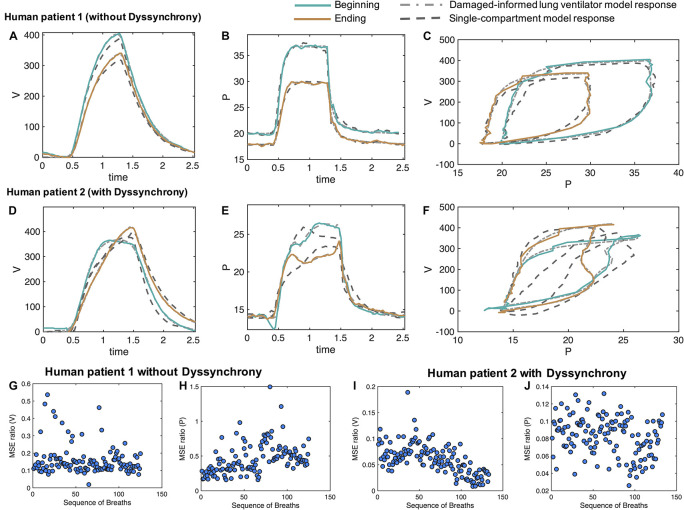
Damaged-informed lung ventilator model can accurately follow breaths of the ICU patients with ARDS without and with dyssynchrony. Measured representative breaths of **(A–C)** patient 1 without dyssynchrony and **(D–F)** patient 2 with flow limited dyssynchrony are shown in solid lines, while the DILV model inferred response is shown in the dashed-dot lines. The response of the single-compartment model is shown in dashed-dashed lines. Histograms of the initial guesses and estimated parameters are shown in the Supplementary Figure 8A. For all the breaths in each case, the corresponding ratio of mean squares errors (MSE) between the DILV model and the single-compartment model is shown in panels **(G–J)**, respectively, while histograms are shown in Supplementary Figures 9, 10. The response of the DILV model was determined using Eqs 1–18 to generate the best-fit model response using estimated mean parameter values shown in Table 1 and Supplementary Table 1. All the data shown here were collected in human-triggered mode (see “Materials and Methods”).

For patient 1, we selected a sequence of 130 breaths without VD at a random location out of 1,829 breaths and performed parameters estimation breath by breath. The sequence of breaths starts at PEEP = 20 cmH_2_O and switches to PEEP = 18 cmH_2_O at breath number 68. Figures 6A–C shows two representative breaths along with the DILV model response at the optimum parameter values (Table 2). Histograms of the initial guesses and estimated parameters are shown in the Supplementary Figure 8A for the respective breaths. For all the model parameters, we observed unimodal estimated parameter distributions with low variance, suggesting that each parameter controls a specific feature of the waveform. We also estimated each breath using the single-compartment model and substantially higher MSE compared to the DILV model (Figures 6G,H). Note that in Figures 6G–J, an MSE ratio less than one indicates that the DILV model produced waveforms that were more similar to the measured data.

Pressure-volume (PV) loops for these cases (Figure 6C) suggest that lung compliance is increased by the prescribed change in PEEP. We found that the model-estimated parameters indicate an increase in compliance (the *A*_*v*_/*A*_*p*__1_ ratio) with a reduction in PEEP (Table 2). The increased compliance at lower PEEP agrees with the single-compartment model (Bates, 2009) response (Supplementary Figures 8B,C) and also the patient outcome (successful extubation). Moreover, a significant increase in parameters *a*_3_ and β_3_ suggested the same (Table 2). The prescribed reduction in PEEP was reflected in a reduction in *A*_*p*__4_.

#### Intensive Care Unit Data-Driven Verification in the Presence of Ventilator Dyssynchrony (Patient 2)

In the cases where VD is present a more thorough parameter interpretation is needed to quantify and understand the patient-ventilator interaction. To show this, from patient 2’s data we randomly selected 133 breaths out of 3,201 breaths that showed mild to severe flow limited VD. Two representative breaths for this case are shown in Figures 6D–F along with the DILV model’s estimate of these breaths at the optimum parameter values (Supplementary Table 1) and the single compartment model fit. Histograms of the initial guesses and estimated parameters for these breaths are shown in the Supplementary Figure 9A.

PV loops for these breaths (Figure 6F) suggest that lung compliance is increased prior to extubation compared to earlier in the ICU course. This qualitative observation is supported by the increased *A*_*v*_/*A*_*p*__1_ ratio (Supplementary Table 1) that is a measure of compliance. However, it is important to note that this compliance includes the additional effects of VD. To further demonstrate the importance of the DILV model, we compared the DILV model’s estimate of the breaths with the single-compartment model (Bates, 2009) estimates for each breath. The single-compartment model estimates of the breaths have substantially higher MSE values for patient 2 compared to patient 1 (Figures 6G–J) due to the presence of VD. Accordingly, we expect large errors in the compliance values estimated with the single-compartment model (Supplementary Figures 9B,C). These results agree with the fact that the DILV model can estimate the volume and pressure waveform data more accurately, especially when the waveforms have high variability, as in the case of patient 2 with VD.

The mouse data verification showed that the DILV model is able to estimate most of the parameters for individual breaths. It is also important to quantify uncertainty across many breaths and in different patients. Analyzing the variability in the estimated parameters over several breaths might capture the lung condition’s heterogeneous nature, including many potentially different and differently damaging breaths that show VD. In the ICU, there are no controlled experiments, and patients simultaneously experience many types and severities of VD, different interventions, heterogeneous injurious insults, etc. As such, we expect to see minor variability in parameter estimates when many breaths of a patient are approximately the same compared to large variability in the parameter estimates when breaths are heterogeneous. For example, we observed a low variability in all the volume model parameters over several breaths with the exception of *A*_*v*_ for patient 1, indicating that the volume waveforms’ characteristic shape remains the same at different time points except for variations in tidal volume. This contrasts with what we observed for patient 2 where VD drove heterogeneity and substantial deviations from more normal breaths (Supplementary Figures 10, 11). More importantly, parameters associated with patient-VD can be used to infer the presence of VD. In patient 2, we observed increases in the β_5_, β_6_, and *A*_*p*__2_ parameters which align with the visual determination of VD in those breaths (Supplementary Figures 10, 11).

Taken together, these results suggest that the DILV model can reproduce a wide variety of waveform data and is capable of extracting hypothesis-driven, clinically-relevant information from the waveforms that might facilitate a systematic interpretation of the dynamics of the injured lung.

## Discussion

In this work, we developed a new damage-informed lung ventilator model that represents pressure and volume waveform data by reconstructing the waveforms from hypothesis-driven modular subcomponents. We then preformed a proof-in-principle verification that the model can potentially represent hypothesized damage in humans and mice. The model accurately estimated pressure and volume waveforms data and consistently distinguished healthy from injured lungs based on parameter estimation. Furthermore, we directly incorporate clinical and physiologic knowledge regarding important and observable features into the model that might be associated with the lung pathophysiology—the subcomponents of the model represent hypothesis-driven deviations from normal breaths. We also analytically define lung compliance in terms of model parameters and demonstrate changes in compliance values that agree with experimentally induced lung injury. This is a proof of principle work where our objective was to develop a ventilator waveform data-based lung model and demonstrate that the model has the potential to be used both with the laboratory and clinical data and infer lung condition.

To demonstrate what is gained with this novel approach, we present a comprehensive comparison between the DILV model and the single-compartment model for a wide range of ventilator waveforms related to different lung conditions and patient-ventilator interactions. We include pressure- and volume-controlled ventilation in healthy and lung-injured mice and in humans in the absence and presence of VD. Through this comparison, we establish that the DILV model can reproduce the features present in the waveform data and report lung compliance values that agree with lung condition (Figures 5, 6 and Supplementary Figures 6–11). This is primarily possible because of our unique waveform-based approach that enabled the model to have enough flexibility. At the same time, the model is limited using prior knowledge so as not to have the capability to estimate every possible variation in PV waveforms, but rather is constrained to estimate the features of the ventilator data that are the most clinically impactful. This approach lives between a machine learning approach, where the model is flexible enough to estimate every feature and must then discern which features are important through regularization to prevent overfitting, and the fully mechanistic lung modeling approach where the observed physiology must emerge from the proposed lung mechanics. It is possible that taking this middle path will help advance all approaches.

The most direct application of the DILV modeling approach is to quantify the qualitative physiological interpretation of pressure and volume data. An experienced clinician or physiologist can infer the status of a patient, the safety of ongoing ventilation, the presence of VD, and other important details from visual inspection. However, we currently do not yet have methods to identify all of these characteristics in ventilator data quantitatively. The entire waveform may be utilized and this provides a rich repository of data that is challenging and time-consuming to use for diagnosis and treatment. In contrast, summarizing the waveform data in scalar values for resistance and compliance may cast aside important details such as identifying dyssynchrony. Our approach may offer a methodology for condensing the pressure-volume data to track changes in injury severity over time, and estimate injury phenotypes (Supplementary Figures 10, 11). A similar phenotype study on a large dataset could be used to categorize and understand lung injury, serve as outcome measures for interventions, and describe the impacts of dyssynchrony (Sottile et al., 2018a) and VILI (Chiumello et al., 2016; Aoyama et al., 2018).

Lung injury diagnosis and decision-making are based in part on the interpretation of the pressure, volume, and flow waveforms. However, different pathophysiologic mechanisms can lead to the same observed waveform features. For example, increased driving pressure could be a result of derecruitment (alveolar collapse) or alveolar flooding (Gattinoni et al., 1987; Smith et al., 2020). In other words, the human-based inference using limited waveform data can be ill-posed. The DILV modeling and parameter estimation approach could allow to estimate a large number of breaths efficiently with unique solutions (Supplementary Figures 10, 11). We could therefore use the DILV model to estimate over many similar but varied breaths, and might be able to better triangulate the most probable pathophysiologic drivers because the primary driver of damage will likely be present, and significant, despite inter-breath variations. At the same time, more extraneous details will not be consistently expressed in every breath.

Note that in the DILV model, an explicit coupling between pressure and volume signals is absent. We have intentionally taken this approach to preserve flexibility so that we can accurately recapitulate a wide variety of clinically and experimentally observed features in the pressure and volume signals, including the effects of VD (Figures 5F,G, 6D–F). Such flexibility in the model outcome is not always possible with rigid coupling between pressure and volume data, as we have shown by comparing the DILV model response with the single-compartment model. This is not to say that pressure and volume are totally independent in the DILV model because we utilize the same respiratory rate for both. In addition, we show that the ratio of a volume and pressure model parameter (*A*_*v*_ and *A*_*p*__1_) describes lung compliance. In future studies, we will link additional specific components of the pressure and volume waveforms through physiologically relevant parameters such as the nonlinearity of lung elastance or inspiratory and expiratory flow resistance. Alternatively, a compartment-based model could be used to incorporate the physiologic coupling between pressure and volume data by utilizing the outputs from the DILV model presented here as inputs for compartment models. If the DILV model is used to preprocess the data before analysis using a compartment model, it is possible to formulate the problem entirely of ordinary differential equations, opening up a range of more efficient inference machinery (Bertsimas and Tsitsiklis, 1997; Nocedal, 2006; Albers et al., 2019b).

Finally, our work here has several notable limitations. *First*, this is a model development work, where we built a new lung model and showed that the model can accurately recapitulate waveforms and estimate accepted physiological parameters such as compliance as a baseline evaluation. This is sufficient for proof-in-principle that the model can capture physiologic differences. However, establishing that the model can accurately differentiate more specifically defined phenotypes will require validation on much larger laboratory and clinical populations. Moreover, to tie the model outputs to injury phenotypes, the pathogenesis of VILI, etc., we will have to establish the concordance between the model estimates of human and mouse-model data in similar settings and validate the phenotypes, etc., within the mouse lung. This work will be done in the following steps: hypothesize damage-related feature(s) within human ventilator, estimate model for humans and extract contextual clinical information (e.g., lung injury source), create mouse model data with a context similar to the human data, estimate the model for mice, validate concordance of model estimates between humans and mice, validate the damage severity and cause within the mice.

*Second*, the human data in this study were collected using a specific ventilator (Hamilton G5) operated in a pressure-controlled volume targeted mode. For the wider applicability of the model, additional data verification is required across different ventilators and ventilation modes. *Third*, in this work we did not explicitly relate our model parameters with the physiological or morphometric data. We rather pose hypotheses about the presence and physiological meaning of waveform features that deviate from normal instead of observing the deviant features emerging from lung physiology. For this interpretation, we relied on the expertise of a laboratory ventilation expert (BS) and a critical care physician (PS). However, it is likely that differing opinions will exist among experts. Collecting and synthesizing such information will require a different qualitative study. Moreover, there may be differing opinions regarding what should and should not be included in the model. This does not negate our novel methodology or the DILV model. In fact, the model was constructed with these issues in mind to be flexible, allowing for the testing of differing hypotheses mind. These issues suggest future work is necessary to understand better and verify clinically important features. Alternatively, we may instead seek to link model features to patient outcomes, thus establishing the important characteristics of the model by linking those parameters to outcomes. *Fourth*, in this manuscript, we have incorporated the human waveform data that has flow limited VD to demonstrated that the DILV has enough flexibility to recapitulate waveforms that have patient-VD. In order to identify and phenotype different types of VD, further model update and evaluation will be required, and this work in progress.

In summary, we developed a data-driven lung and ventilator model that can reproduce the commonly observed features in pressure and volume waveforms during mechanical ventilation. The performance of the model was verified with experimental and clinical data in healthy and injured lungs to demonstrate model efficacy in robustly estimating parameters. These parameters are hypothetically linked to both physiologic and ventilator-based mechanisms. Furthermore, the model outputs yield a pulmonary system compliance that is in good agreement with single compartment model estimates and, as expected, compliance decreases with acute lung injury. This methodology represents a departure from many lung modeling efforts, and suggests future directions of work that can provide another pathway for better understanding lung function during mechanical ventilation and can potentially form a bridge between experimental physiology and clinical practice.

## Data Availability Statement

The original contributions presented in the study are included in the article/Supplementary Material, further inquiries can be directed to the corresponding authors.

## Ethics Statement

The studies involving human participants were reviewed and approved by Colorado Multiple Institutional Review Board. The Ethics Committee waived the requirement of written informed consent for participation. The animal study was reviewed and approved by University of Colorado, Anschutz Medical Campus Institutional Animal Care Committee (IACUC)-approved protocol (#00230).

## Author Contributions

DKA, BS, and DJA conception and design of the research, analyzed the data, and evaluated the model. DJA and DKA developed the mathematical model. DKA, BS, and PS designed and conducted the experiments. DKA prepared the figures and wrote the original draft of the manuscript. DKA, BS, PS, and DJA interpreted the results, edited and revised the manuscript, and approved the final version of the manuscript.

## Conflict of Interest

The authors declare that the research was conducted in the absence of any commercial or financial relationships that could be construed as a potential conflict of interest.

## Publisher’s Note

All claims expressed in this article are solely those of the authors and do not necessarily represent those of their affiliated organizations, or those of the publisher, the editors and the reviewers. Any product that may be evaluated in this article, or claim that may be made by its manufacturer, is not guaranteed or endorsed by the publisher.
